# Serial lung ultrasounds in pediatric pneumonia in Mozambique and Pakistan

**DOI:** 10.1038/s41598-021-85485-y

**Published:** 2021-03-18

**Authors:** Amy Sarah Ginsburg, Imran Nisar, Lola Madrid, Jennifer L. Lenahan, Benazir Balouch, Pio Vitorino, Jun Hwang, Alessandro Lamorte, Neel Kanth, Rubao Bila, Marta Valente, Rosauro Varo, Susanne May, Quique Bassat, Fyezah Jehan, Giovanni Volpicelli

**Affiliations:** 1 University of Washington, Seattle, WA USA; 2 Department of Pediatrics and Child Health, Aga Khan University, Karachi, Pakistan; 3 ISGlobal, Hospital Clínic - Universitat de Barcelona, Barcelona, Spain; 4 Save the Children Federation, Inc, Seattle, WA USA; 5 Centro de Investigação Em Saúde de Manhiça (CISM), Maputo, Mozambique; 6 Department of Emergency Medicine, Parini Hospital, Aosta, Italy; 7 Sindh Government Children’s Hospital-Poverty Eradication Initiative, Karachi, Pakistan; 8 Department of Emergency Medicine, San Luigi Gonzaga University Hospital, Orbassano, Italy; 9 University of Washington Clinical Trial Center, Building 29, Suite 250, 6200 NE 74th Street, Seattle, WA 98115 USA

**Keywords:** Diseases, Health care, Medical research

## Abstract

Lung ultrasound (LUS) is a promising point-of-care imaging technology for diagnosing and managing pneumonia. We sought to explore serial LUS examinations in children with chest-indrawing pneumonia in resource-constrained settings and compare their clinical and LUS imaging courses longitudinally. We conducted a prospective, observational study among children aged 2 through 23 months with World Health Organization Integrated Management of Childhood Illness chest-indrawing pneumonia and among children without fast breathing, chest indrawing or fever (no pneumonia cohort) at 2 district hospitals in Mozambique and Pakistan. We assessed serial LUS at enrollment, 2, 6, and 14 days, and performed a secondary analysis of enrolled children’s longitudinal clinical and imaging courses. By Day 14, the majority of children with chest-indrawing pneumonia and consolidation on enrollment LUS showed improvement on follow-up LUS (100% in Mozambique, 85.4% in Pakistan) and were clinically cured (100% in Mozambique, 78.0% in Pakistan). In our cohort of children with chest-indrawing pneumonia, LUS imaging often reflected the clinical course; however, it is unclear how serial LUS would inform the routine management of non-severe chest-indrawing pneumonia.

## Introduction

Pneumonia remains the leading infectious killer of children, particularly in resource-constrained settings^1^. Effective and timely diagnosis and management are critical to saving lives. Given the frequent lack of imaging capacity in resource-constrained settings and restrictions on use of imaging modalities with ionizing radiation in pediatric populations, much remains unknown about what takes place in the lungs of children with chest-indrawing pneumonia undergoing antibiotic treatment in resource-constrained settings. Lung ultrasound (LUS) is a portable, point-of-care tool that can dynamically visualize lungs at the bedside with promising diagnostic accuracy for pneumonia and greater sensitivity or specificity when compared with chest radiography (CXR)^2–5^. LUS can be used to visualize the lungs multiple times over the course of a child’s illness without risk of exposing the child to ionizing radiation. We conducted a pilot study in Mozambique and Pakistan to investigate the use of LUS for diagnosis among children with World Health Organization (WHO) Integrated Management of Childhood Illness (IMCI) chest-indrawing pneumonia and found that expert LUS interpreters may achieve substantially higher interrater reliability (IRR) for LUS compared to CXR^6^. In this secondary analysis, we explore serial LUS examinations in children with chest-indrawing pneumonia and compare their clinical and imaging courses longitudinally.

## Methods

### Study design, setting and participants

Methods and primary results of this prospective, observational, facility-based cohort study have been described previously^6, 7^. Secondary and exploratory objectives included tracking and comparing LUS and clinical presentations longitudinally at enrollment, and on Days 2, 6, and 14 among children with and without chest-indrawing pneumonia.

**Figure 1:**
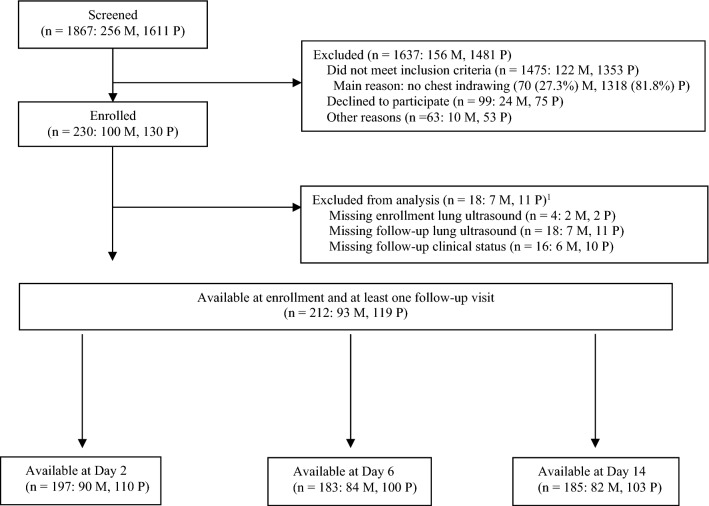
Flow of children with chest-indrawing pneumonia by country: Mozambique (M), Pakistan (P). ^1^Reasons for exclusion from analysis are not mutually exclusive.

Children aged 2 through 23 months meeting WHO IMCI chest-indrawing pneumonia case definition (chest-indrawing pneumonia cohort) in outpatient and/or emergency departments of Manhiça District Hospital in Manhiça, Mozambique and Sindh Government Children’s Hospital–Poverty Eradication Initiative in Karachi, Pakistan were screened by study staff to determine eligibility (Table 1, Fig. 1). We chose to exclude children with more severe pneumonia and disease since we did not believe LUS would change initial management given that the standard of care for these children is to be admitted to hospital and receive empiric intravenous antibiotics. A separate group of 40 children presenting with complaints of cough or difficulty breathing but without fast breathing, chest indrawing or fever (no pneumonia cohort) was also screened. Conducted in accordance with International Conference on Harmonisation, Good Clinical Practice and Declaration of Helsinki 2008, the study was approved by Western Institutional Review Board, Comité Nacional de Bioética para a Saúde (246/CNBS/17), Comite de Ética del Hospital Clínic de Barcelona (HCB/2017/0074), and Aga Khan University Ethics Review Committee, and registered with ClinicalTrials.gov (Registration: NCT03187067; 14/06/2017).

**Table 1:** Study definitions and clinical outcomes.

**Study definitions**	
Chest-indrawing pneumonia	Cough less than 14 days or difficulty breathing AND visible indrawing of the chest wall with or without fast breathing for age
Fast breathing for age	Respiratory rate ≥ 50 breaths per minute (for children 2 to < 12 months of age) or ≥ 40 breaths per minute (for children ≥ 12 months of age)
Very fast breathing for age	≥ 70 breaths per minute (for children 2 to < 12 months of age) or ≥ 60 breaths per minute (for children ≥ 12 months of age)
Hypoxemia	Arterial oxyhemoglobin saturation (SpO_2_) < 90% in room air, as assessed non-invasively by a pulse oximeter
Danger signs	General danger signs: convulsions; lethargy or unconsciousness; inability to drink or feed; vomiting everything; or stridor in calm child
	Respiratory distress: grunting; nasal flaring; head nodding; severe chest indrawing
**Lung ultrasound progressions**	
Improved	Between enrollment and Day 2: decreased number or size(s) ≥ 0.5 cm of consolidation(s), without any increase(s) Between enrollment and Day 6, Day 14: decreased number or size(s) ≥ 1 cm of consolidation(s), without any increase(s)
Stable	Between enrollment and Day 2: no observed changes to number or size(s) ≥ 0.5 cm of consolidation(s) Between enrollment and Day 6, Day 14: no observed change(s) to number or size(s) ≥ 1 cm of consolidation(s)
Worsened	Between enrollment and Day 2: increased number or size(s) ≥ 0.5 cm of consolidation(s) Between enrollment and Day 6, Day 14: increased number or size(s) ≥ 1 cm of consolidation(s)
**Clinical outcomes**	
Clinically cured	Absence of danger signs, hypoxemia, fever, chest indrawing, and very fast breathing
Not clinically cured	Presence of any danger sign, hypoxemia, fever, chest indrawing, or very fast breathing

### Study procedures

After obtaining informed consent from parents and/or legal guardians for study participation and upon enrollment on Day 1, eligible children underwent history, physical and LUS examinations. Enrolled children received local standard of care without the results of LUS examinations informing clinical care.

On enrollment and Days 2, 6, and 14, LUS examinations (longitudinal and oblique videos obtained of the anterior, lateral and posterior sides of the chest) were performed by 4 trained non-physician healthcare personnel (nurse and medical agent in Mozambique, and 2 radiology technicians in Pakistan) who received 1-day standardized training course and 3-day supervised practice. LUS interpretation using a standardized scoresheet targeted detection of typical lung consolidations, pleural effusions, interstitial patterns and obstructive atelectasis^7, 8^. At least 2 independent physicians expertly trained in LUS interpretation and blinded to clinical presentation interpreted each LUS examination. If discordant, a designated expert LUS interpreter acted as arbiter. LUS operators at each site also interpreted LUS examinations, independently from one another. All interpretations were performed in batches at a later time using the same standardized scoresheet.

Blood samples were tested for hemoglobin and C-reactive protein in Mozambique and Pakistan, and for procalcitonin, malaria and HIV in Mozambique.

### Statistical analysis

LUS progression (improved vs stable vs worsened) is defined on Days 2, 6, and 14 by comparing the number and size(s) of consolidations in each respective follow-up LUS to either the enrollment or last available LUS (Table 1). Chi-squared and Fisher’s exact tests were used to compare categorical variables between groups while t-tests were used to compare continuous variables. Tests were 2-sided using an alpha-level of 0.05. No adjustments for multiple comparisons were made, given the exploratory nature of the analyses. All analyses were performed using R (version 3.6.3; R Foundation for Statistical Computing)^9^.

## Results

The chest-indrawing pneumonia cohort enrolled 100 and 130 children from Mozambique and Pakistan, respectively. Of the LUS and clinical data for these children, 90 and 110 were available at Day 2, 84 and 100 at Day 6, and 82 and 103 at Day 14, from Mozambique and Pakistan, respectively (Fig. 1). Children in the chest-indrawing pneumonia cohort with enrollment and at least 1 follow-up LUS enrolled in Pakistan were generally younger than those in Mozambique, with a lower proportion of female children (39.8% in Mozambique, 26.1% in Pakistan; Table 2). Higher C-reactive protein levels and lower average oxyhemoglobin saturation suggest that severity of disease may be greater in Pakistan, though average hemoglobin level was slightly lower in Mozambique. More children from Pakistan had consolidations on LUS at enrollment than those from Mozambique (42.9% vs 14.0%, p < 0.01).

**Table 2:** Baseline characteristics of enrolled and analyzed^1^ children in the chest-indrawing pneumonia cohort by country.

	Mozambique	Pakistan	Between-country comparison p-value
	(n = 93)	(n = 119)	
**Age (months)**			
Mean (SD)	10.7 (6.0)	6.8 (4.7)	< 0.01
< 12, n (%)	53 (57.0%)	104 (87.4%)	< 0.01
Female, n (%)	37 (39.8%)	31 (26.1%)	0.06
**Temperature (°C)**			
Mean (SD)	37.1 (1.1)	36.7 (0.8)	< 0.01
Fever (≥ 38 °C), n (%)	20 (21.5%)	10 (8.4%)	0.01
**Respiratory rate (breaths per minute)**			
< 12 months, mean (SD)	52.9 (11.4)	53.4 (7.9)	0.74
≥ 12 months, mean (SD)	44.4 (10.2)	47.9 (10.2)	0.26
Fast breathing, n (%)	50 (53.8%)	75 (63.6%)	0.18
Oxyhemoglobin saturation (%), mean (SD)	97.8 (2.7)	95.3 (2.2)	< 0.01
< 90%, n (%)	1 (1.1%)	0 (0.0%)	0.44
Hemoglobin (g/dL), mean (SD)	10.0 (1.3)	10.7 (1.3)	< 0.01
Positive HIV rapid diagnostic test, n (%)^2^	0 (0.0%)		
Positive malaria rapid diagnostic test, n (%)^3^	1 (1.1%)		
C-reactive protein (ug/mL), mean (SD)^4^	38.9 (51.0)	103.3 (192.1)	< 0.01
Procalcitonin (ng/mL), mean (SD)^5^	1.7 (7.4)		
Consolidation on lung ultrasound, n (%)	13 (14.0%)	51 (42.9%)	< 0.01

*SD* standard deviation.

^1^Baseline characteristics of enrolled children with at least 1 follow-up visit on Day 2, 6, or 14.

^2^HIV testing only conducted in Mozambique; missing or indeterminate for 54 children.

^3^Malaria testing only conducted in Mozambique; missing for 2 children.

^4^C-reactive protein missing for 5 children in Mozambique.

^5^Procalcitonin only measured in Mozambique; missing for 5 children.

The no pneumonia cohort enrolled 20 children from Mozambique and 20 children from Pakistan. Of note, only 2 children in the no pneumonia cohort, both from Pakistan, had consolidations on any LUS: 1 child on enrollment but not follow-up LUS and 1 child on Day 2 LUS (data not shown). Additionally, 1 child’s data was not available because no visits were recorded beyond enrollment. The remaining 37 available LUS and clinical data for children in the no pneumonia cohort in Mozambique and Pakistan had normal enrollment and follow-up LUS examinations. We restrict our attention to the chest-indrawing pneumonia cohort for the remainder of the results.

When comparing each follow-up LUS directly to the enrollment LUS, many general progression categories appeared with similar frequency between countries (Tables 3 and 4). Among children with consolidations on enrollment LUS, more than half of the follow-up LUS improved on Day 2 (58.3% in Mozambique, 60.9% in Pakistan), while roughly a third worsened (33.3% in Mozambique, 34.8% in Pakistan), and a smaller number remained stable (8.3% in Mozambique, 4.3% in Pakistan, p = 0.76). By Day 6, only a quarter of follow-up LUS worsened or remained stable rather than improved (76.9% improved and 23.1% worsened in Mozambique, 75.0% improved, 13.6% worsened, and 11.4% stable in Pakistan, p = 0.42). By Day 14, the majority of follow-up LUS had improved (100% improved in Mozambique, 85.4% improved, 9.8% worsened, 4.9% stable in Pakistan, p = 0.73). Among children without consolidations on enrollment LUS, the vast majority of children’s follow-up LUS remained stable rather than worsened (Day 2: 92.3% in Mozambique, 90.6% in Pakistan, p = 0.77; Day 6: 93.0% in Mozambique, 94.6% in Pakistan, p > 0.99; Day 14: 97.2% in Mozambique, 98.4% in Pakistan, p > 0.99).

**Table 3:** Progression of consolidation on lung ultrasound (LUS) by Days 2, 6 and 14 clinical status in Mozambique.

General LUS progression category from enrollment to:	Chest-indrawing pneumonia cohort: Mozambique					
	Consolidation on enrollment LUS			No consolidation on enrollment LUS		
	Clinically cured	Not clinically cured	Overall	Clinically cured	Not clinically cured	Overall
Day 2	(n = 4)	(n = 8)	(n = 12)	(n = 24)	(n = 54)	(n = 78)
Improved^1,2^	2 (50.0%)	5 (62.5%)	7 (58.3%)	–	–	–
Stable^1,3^	0 (0.0%)	1 (12.5%)	1 (8.3%)	22 (91.7%)	50 (92.6%)	72 (92.3%)
Worsened ^1,4^	2 (50.0%)	2 (25.0%)	4 (33.3%)	2 (8.3%)	4 (7.4%)	6 (7.7%)
Day 6	(n = 8)	(n = 5)	(n = 13)	(n = 50)	(n = 21)	(n = 71)
Improved^1,5^	7 (87.5%)	3 (60.0%)	10 (76.9%)	–	–	–
Stable^1,6^	0 (0.0%)	0 (0.0%)	0 (0.0%)	48 (96.0%)	18 (85.7%)	66 (93.0%)
Worsened ^1,7^	1 (12.5%)	2 (40.0%)	3 (23.1%)	2 (4.0%)	3 (14.3%)	5 (7.0%)
Day 14	(n = 11)	(n = 0)	(n = 11)	(n = 61)	(n = 10)	(n = 71)
Improved^1,5^	11 (100.0%)	–	11 (100.0%)	–	–	–
Stable^1,6^	0 (0.0%)	–	0 (0.0%)	60 (98.4%)	9 (90.0%)	69 (97.2%)
Worsened^1,7^	0 (0.0%)	–	0 (0.0%)	1 (1.6%)	1 (10.0%)	2 (2.8%)

^1^Percentage of children with known progression category.

^2^Comparison of enrollment and Day 2 LUS showing decreased number or size(s) ≥ 0.5 cm of consolidation(s), without any increase(s).

^3^Comparison of enrollment and Day 2 LUS showing no observed changes to number or size(s) ≥ 0.5 cm of consolidation(s).

^4^Comparison of enrollment and Day 2 LUS showing increased number or size(s) ≥ 0.5 cm of consolidation(s).

^5^Comparison of enrollment and Day 6 or Day 14 (as applicable) LUS showing decreased number or size(s) ≥ 1 cm of consolidation(s), without any increase(s).

^6^Comparison of enrollment and Day 6 or Day 14 (as applicable) LUS showing no observed changes to number or size(s) ≥ 1 cm of consolidation(s).

^7^Comparison of enrollment and Day 6 or Day 14 (as applicable) LUS showing increased number or size(s) ≥ 1 cm of consolidation(s).

**Table 4:** Progression of consolidation on lung ultrasound (LUS) by Days 2, 6 and 14 clinical status in Pakistan.

General LUS progression category from enrollment to:	Chest-indrawing pneumonia cohort: Pakistan					
	Consolidation on enrollment LUS			No consolidation on enrollment LUS		
	Clinically cured	Not clinically cured	Overall	Clinically cured	Not clinically cured	Overall
Day 2	(n = 5)	(n = 41)	(n = 46)	(n = 2)	(n = 62)	(n = 64)
Improved^1,2^	2 (40.0%)	26 (63.4%)	28 (60.9%)	–	–	–
Stable^1,3^	0 (0.0%)	2 (4.9%)	2 (4.3%)	2 (100.0%)	56 (90.3%)	58 (90.6%)
Worsened^1,4^	3 (60.0%)	13 (31.7%)	16 (34.8%)	0 (0.0%)	6 (9.7%)	6 (9.4%)
Day 6	(n = 26)	(n = 18)	(n = 44)	(n = 36)	(n = 20)	(n = 56)
Improved^1,5^	21 (80.8%)	12 (66.7%)	33 (75.0%)	–	–	–
Stable^1,6^	4 (15.4%)	1 (5.6%)	5 (11.4%)	34 (94.4%)	19 (95.0%)	53 (94.6%)
Worsened ^1,7^	1 (3.8%)	5 (27.8%)	6 (13.6%)	2 (5.6%)	1 (5.0%)	3 (5.4%)
Day 14	(n = 32)	(n = 9)	(n = 41)	(n = 54)	(n = 8)	(n = 62)
Improved^1,5^	30 (93.8%)	5 (55.6%)	35 (85.4%)	–	–	
Stable^1,6^	1 (3.1%)	1 (11.1%)	2 (4.9%)	54 (100.0%)	7 (87.5%)	61 (98.4%)
Worsened^1,7^	1 (3.1%)	3 (33.3%)	4 (9.8%)	0 (0.0%)	1 (12.5%)	1 (1.6%)

^1^Percentage of children with known progression category.

^2^Comparison of enrollment and Day 2 LUS showing decreased number or size(s) ≥ 0.5 cm of consolidation(s), without any increase(s).

^3^Comparison of enrollment and Day 2 LUS showing no observed changes to number or size(s) ≥ 0.5 cm of consolidation(s).

^4^Comparison of enrollment and Day 2 LUS showing increased number or size(s) ≥ 0.5 cm of consolidation(s).

^5^Comparison of enrollment and Day 6 or Day 14 (as applicable) LUS showing decreased number or size(s) ≥ 1 cm of consolidation(s), without any increase(s).

^6^Comparison of enrollment and Day 6 or Day 14 (as applicable) LUS showing no observed changes to number or size(s) ≥ 1 cm of consolidation(s).

^7^Comparison of enrollment and Day 6 or Day 14 (as applicable) LUS showing increased number or size(s) ≥ 1 cm of consolidation(s).

Another way to categorize the LUS trajectories is to compare each follow-up LUS to the last, previously available LUS, and to judge the trajectory as improved or worsened by Day 14 based on the last such change, or as stable if no changes were observed. Among the LUS trajectories categorized this way for children with consolidations on enrollment LUS, by Day 14 100% of children’s LUS improved in Mozambique, while 78.0% improved, 2.4% remained stable, and 19.5% worsened in Pakistan (Appendix 1). Among the LUS trajectories categorized this way for children without consolidations on enrollment LUS from Mozambique and Pakistan, respectively, by Day 14, 9.9% and 11.3% of children’s follow-up LUS improved (after previously worsening), 1.4% and 1.6% worsened, and 88.7% and 87.1% were stable (i.e., remained without consolidation).

While a minority of the children appeared clinically cured on Day 2 (31.1% in Mozambique, 6.4% in Pakistan), a majority of children appeared clinically cured by Day 6 (69.0% in Mozambique, 62.0% in Pakistan) and by Day 14 (87.8% in Mozambique, 83.5% in Pakistan) (Appendix 2).

Among children with consolidations on enrollment LUS, associations between clinically cured status (yes vs no) and LUS progression categories (improved, stable, and worsened) were not statistically significant, except for in Pakistan on Day 14 where improvement on follow-up LUS was seen in 93.8% of children who were clinically cured and 55.6% who were not clinically cured (p = 0.01) (Tables 3 and 4). By Day 14, the majority of children with chest-indrawing pneumonia and consolidation on LUS were clinically cured (100% in Mozambique, 78.0% in Pakistan).

Among children without consolidations on enrollment LUS, a similar proportion of follow-up LUS on Day 2 worsened in those who were clinically cured compared to those who were not in Mozambique (8.3% vs 7.4%, p > 0.99). In Pakistan, only 2 children without consolidations on enrollment LUS were considered clinically cured on Day 2, with both having stable follow-up LUS; 9.7% of those not clinically cured by Day 2 showed worsening on follow-up LUS (0% vs 9.7%, p > 0.99). By Day 6, few children without consolidations on enrollment LUS appeared to have worsened on follow-up LUS regardless of whether they appeared clinically cured or not; differences were not statistically significant (4.0% vs 14.3%, p = 0.15 in Mozambique; 5.6% vs 5.0% in Pakistan, p > 0.99). By Day 14, among children with no consolidation on enrollment LUS, there were no statistically significant differences in progression of LUS patterns by clinically cured category (1.6% vs 10.0%, p = 0.26 in Mozambique; 0% vs 12.5%, p = 0.13 in Pakistan), with 1 or no child appearing to worsen on follow-up LUS in each category for each country.

## Discussion

In the first study of its kind to longitudinally follow children with chest-indrawing pneumonia in resource-constrained settings with serial LUS examinations, we sought to characterize and track the trajectory of the children’s pneumonias through LUS imaging. Other published reports describe using serial LUS in adults in high-resource settings to monitor lung involvement, disease progression, and treatment response during management of pneumonia, avian influenza A (H7N9) respiratory failure, and most recently, COVID-19 infection^10–14^. In children, serial LUS follow-up has been used in high-resource settings for the diagnosis and management of bronchiolitis, pneumonia, and COVID-19 infection^15–20^.

In our study following a chest-indrawing pneumonia cohort in both Mozambique and Pakistan with serial LUS, the majority of the children did not show consolidations on their enrollment LUS. For those who did show consolidation on their enrollment LUS, 88.5% showed improvement on follow-up LUS by Day 14 and 82.7% were clinically cured by Day 14. By Day 14, most children in the chest-indrawing pneumonia cohort were clinically cured (87.8% in Mozambique, 83.5% in Pakistan) regardless of whether their imaging worsened on follow-up LUS from enrollment LUS. Of note, 1 out of 2 (50.0%) children in Mozambique who appeared to worsen on follow-up LUS were clinically cured; 1 out of 5 (20.0%) children in Pakistan who appeared to worsen on follow-up LUS were clinically cured. Based on these results, it is unclear how serial LUS would impact the routine management of non-severe chest-indrawing pneumonia.

In our cohort of children with chest-indrawing pneumonia, the LUS imaging often reflected the clinical course; however, there were some differences. On Day 2, LUS appeared to lag behind the clinical presentation with children appearing worse on follow-up LUS despite appearing clinically cured ((2 + 2)/(4 + 24) = 14.3% in Mozambique; (3 + 0)/(5 + 2) = 42.9% in Pakistan), but by Day 6 when the 5-day antibiotic treatment course is typically completed, LUS still appeared to lag behind in a smaller minority ((1 + 2)/(8 + 50) = 5.2% in Mozambique; (1 + 2)/(26 + 36) = 4.8% in Pakistan). Yet, by Day 14, with few exceptions ((0 + 1)/(11 + 61) = 1.4% in Mozambique and (1 + 0)/(32 + 54) = 1.2% in Pakistan), LUS corresponded with the clinical presentation. Conventionally, imaging is thought to lag behind clinical presentation and is not necessarily concordant with clinical cure^21^. Despite this lag, we may have anticipated that a higher percentage of children who were clinically cured would show improvement on follow-up LUS compared to those who were not clinically cured, or conversely, that a higher proportion of children not clinically cured would remain stable or worsen on follow-up LUS. Most of the data in Mozambique and Pakistan were consistent with this expectation but did not reach statistical significance because of small numbers. The only exception was Day 14 in Pakistan among those with consolidation on enrollment LUS; the vast majority (93.8%) of those clinically cured showed LUS improvement whereas only about half (55.6%) among those not clinically cured showed LUS improvement. Because changes in LUS were judged in a relative manner, an improvement on LUS did not necessarily equate to cure.

While obtaining serial LUS does not appear to add significant value to the routine management of non-severe chest-indrawing pneumonia, our study was constrained by the relatively small sample sizes of enrolled children. Furthermore, limited comparisons could be made between sites and between pneumonia and no pneumonia cohorts due to the differing study populations between Mozambique and Pakistan. Worth noting, 81.8% in Pakistan vs 27.3% in Mozambique of children screened were not eligible for study enrollment because they did not exhibit chest indrawing. While care was taken to make certain that all eligibility criteria were confirmed at both study sites, this difference in screening numbers could be due to differences in care-seeking behavior at the study sites and/or differences in screening procedures. Also likely contributing to the larger differences in those screened vs enrolled in Pakistan, was that far more children presented to the study site in Pakistan than in Mozambique, prompting faster enrollment in the pneumonia cohort in Pakistan. Lower average oxyhemoglobin saturations, higher C-reactive protein measurements, more LUS consolidation and interstitial patterns, and somewhat poorer LUS trajectories by Day 14 in Pakistan suggest that sicker children may have been enrolled in Pakistan compared to Mozambique. These differences may be reflected in the results for Day 14, by which time all children in Mozambique who had LUS consolidations at enrollment had no evidence of disease on follow-up LUS or clinically.

Other study limitations include our analysis of disease progression on LUS for which we evaluated each follow-up LUS in comparison to a previous LUS, rather than the absolute presence or absence of disease at each timepoint. Additionally, our study was limited by the small proportion of children who clinically did not do well despite antibiotic treatment. Because so few children in the chest-indrawing pneumonia cohort were not clinically cured by Day 14, it is difficult to determine whether LUS imaging can help inform management of uncomplicated chest-indrawing pneumonia in children.

In our pilot study in Mozambique and Pakistan, following children with chest-indrawing pneumonia longitudinally with serial LUS did not appear to suggest a significant added value to management. It is important to remember that imaging is a part of the clinical assessment of pneumonia and should not be taken out of context of the child’s clinical presentation. Use of serial LUS may be more helpful in the management of those with more severe disease or those with comorbidities.

## Supplementary Information

Supplementary Information.

## Data Availability

All data generated or analyzed for this study are included in this published article.
