# Supplementary material for: Serial lung ultrasounds in pediatric pneumonia in Mozambique and Pakistan
Source: Sci Rep. 2021 Mar 18;11:6262. doi: 10.1038/s41598-021-85485-y (PMC7973793; doi:10.1038/s41598-021-85485-y)
Supplement: Supplementary file 1 — Supplementary Information. [file 41598_2021_85485_MOESM1_ESM.docx]

# **Title:** Serial lung ultrasounds in pediatric pneumonia in Mozambique and Pakistan

**Authors:**  Amy Sarah Ginsburg,^1^* Imran Nisar,^2^ Lola Madrid,^3^ Jennifer L Lenahan,^4^ Benazir Balouch,^2^ Pio Vitorino,^5^ Jun Hwang,^1^ Alessandro Lamorte,^6^ Neel Kanth,^7^ Rubao Bila,^5^ Marta Valente,^3^ Rosauro Varo,^3^ Susanne May,^1^ Quique Bassat,^3^ Fyezah Jehan,^2^ Giovanni Volpicelli^8^

^1^ University of Washington, Seattle, WA, USA

^2^ Department of Pediatrics and Child Health, Aga Khan University, Karachi, Pakistan

^3^ ISGlobal, Hospital Clínic - Universitat de Barcelona, Barcelona, Spain

^4^ Save the Children Federation, Inc., Seattle, WA, USA

^5^ Centro de Investigação em Saúde de Manhiça (CISM), Maputo, Mozambique

^6^ Department of Emergency Medicine, Parini Hospital, Aosta, Italy

^7^ Sindh Government Children’s Hospital–Poverty Eradication Initiative, Karachi, Pakistan

^8^ Department of Emergency Medicine, San Luigi Gonzaga University Hospital, Orbassano, Italy

* **Corresponding Author**: Amy Sarah Ginsburg, University of Washington Clinical Trial Center Building 29, Suite 250, 6200 NE 74^th^ Street, Seattle, WA, 98115, USA; Phone: 206.543.1044; Email: [messageforamy@gmail.com](mailto:messageforamy@gmail.com)

**Financial Support**: This work was supported by grants from the Bill and Melinda Gates Foundation (OPP1105080) and Save the Children.

**Running Title:**  Serial lung ultrasounds in pediatric pneumonia

**Key Words**: lung ultrasound; pediatric pneumonia; serial; longitudinal; low-resource settings

| **Appendix 1. Progression of consolidation on lung ultrasound (LUS) through Day 14*** | | | | | | | |
| --- | --- | --- | --- | --- | --- | --- | --- |
| Enrollment vs Day 2 | Last observed prior to Day 6 vs Day 6 | Last observed prior to Day 14 vs Day 14 | Mozambique | | Pakistan | | Interpretation |
|  |  |  | Consolidation at enrollment (n = 11) | No consolidation at enrollment (n = 71) | Consolidation at enrollment (n = 41) | No consolidation at enrollment (n = 62) |  |
| Improved | | | 11 (100.0%) | 7 (9.9%) | 32 (78.0%) | 7 (11.3%) | Consolidations improved on Day 14 or were stable on Day 14 after improvement |
| v | - | - | 5 (45.5%) |  | 11 (26.8%) |  | Improved, stable, stable |
| ^ | v | - | 2 (18.2%) | 3 (4.2%) | 2 (4.9%) | 3 (4.8%) | Worsened, improved, stable |
| v | v | - |  |  | 6 (14.6%) |  | Improved, improved, stable |
| ^ | v | v | 1 (9.1%) | 1 (1.4%) | 2 (4.9%) |  | Worsened, improved, improved |
| - | ^ | v |  | 3 (4.2%) |  | 2 (3.2%) | Stable, worsened, improved |
| v | ^ | v |  |  | 4 (9.8%) |  | Improved, worsened, improved |
| ^ | ? | v | 1 (9.1%) |  | 1 (2.4%) | 1 (1.6%) | Worsened, unknown on Day 6, improved on Day 14 |
| ? | ^ | v | 1 (9.1%) |  | 1 (2.4%) |  | Unknown on Day 2, worsened on Day 6 from enrollment, improved |
| - | v | - | 1 (9.1%) |  |  |  | Stable, improved, stable |
| - | - | v |  |  | 1 (2.4%) |  | Stable, stable, improved |
| ? | ? | v |  |  | 1 (2.4%) |  | Unknown on Days 2 and 6, improved on Day 14 |
| ? | v | - |  |  | 1 (2.4%) |  | Unknown on Day 2, improved on Day 6 from enrollment, stable |
| ? | v | v |  |  | 1 (2.4%) |  | Unknown on Day 2, improved on Days 6 and 14 |
| v | ? | v |  |  | 1 (2.4%) |  | Improved, unknown on Day 6, improved on Day 14 |
| ^ | ^ | v |  |  |  | 1 (1.6%) | Worsened, worsened, improved |
| Stable | | | 0 (0.0%) | 63 (88.7%) | 1 (2.4%) | 54 (87.1%) | No improvement or worsening of consolidations observed |
| - | - | - |  | 58 (81.7%) | 1 (2.4%) | 47 (75.8%) | Stable, stable, stable |
| - | ? | - |  | 4 (5.6%) |  | 3 (4.8%) | Stable, unknown on Day 6, stable on Day 14 |
| ? | - | - |  | 1 (1.4%) |  | 3 (4.8%) | Unknown on Day 2, stable on Days 6 and 14 |
| ? | ? | - |  |  |  | 1 (1.6%) | Unknown on Days 2 and 6, stable on Day 14 |
| Worsened | | | 0 (0.0%) | 1 (1.4%) | 8 (19.5%) | 1 (1.6%) | Consolidations worsened on Day 14 or were stable on Day 14 after worsening |
| ^ | v | ^ |  |  | 4 (9.8%) |  | Worsened, improved, worsened |
| ^ | ^ | ^ |  |  | 1 (2.4%) | 1 (1.6%) | Worsened, worsened, worsened |
| - | - | ^ |  | 1 (1.4%) |  |  | Stable, stable, worsened |
| ? | v | ^ |  |  | 1 (2.4%) |  | Unknown on Day 2, improved, worsened |
| v | ? | ^ |  |  | 1 (2.4%) |  | Improved, unknown on Day 6, worsened |
| v | ^ | - |  |  | 1 (2.4%) |  | Improved, worsened, stable |
| * Overall trajectories through Day 14 were considered uncategorizable if the Day 14 LUS was not available | | | | | | | |
| v denotes decreased number of LUS consolidations, or decreased size(s) ≥ 0.5 cm from enrollment to Day 2 or ≥ 1 cm from Day 2 to Day 6 or from Day 6 to Day 14, without any increases | | | | | | | |
| - denotes no changes to the number of LUS consolidations and no changes to size(s) ≥ 0.5 cm from enrollment to Day 2 or ≥ 1 cm from Day 2 to Day 6 or from Day 6 to Day 14 | | | | | | | |
| ? denotes unknown changes to LUS consolidation(s) | | | | |  |  |  |

| **Appendix 2. Longitudinal characteristics of enrolled and analyzed children by country** | | | | | |  |
| --- | --- | --- | --- | --- | --- | --- |
|  | Chest-indrawing pneumonia cohort | | | | | |
|  | Mozambique | | | Pakistan | | |
|  | Day 2 | Day 6 | Day 14 | Day 2 | Day 6 | Day 14 |
|  | (n = 90) | (n = 84) | (n = 82) | (n = 110) | (n = 100) | (n = 103) |
| General danger signs, n (%) | 1 (1.1%) | 1 (1.2%) | 0 (0.0%) | 0 (0.0%) | 0 (0.0%) | 1 (1.0%) |
| Convulsions, n (%) | 1 (1.1%) | 1 (1.2%) | 0 (0.0%) | 0 (0.0%) | 0 (0.0%) | 0 (0.0%) |
| Lethargy, n (%) | 0 (0.0%) | 0 (0.0%) | 0 (0.0%) | 0 (0.0%) | 0 (0.0%) | 1 (1.0%) |
| Unable to drink or feed, n (%) | 0 (0.0%) | 0 (0.0%) | 0 (0.0%) | 0 (0.0%) | 0 (0.0%) | 0 (0.0%) |
| Vomits everything, n (%) | 0 (0.0%) | 0 (0.0%) | 0 (0.0%) | 0 (0.0%) | 0 (0.0%) | 0 (0.0%) |
| Stridor in calm child, n (%) | 0 (0.0%) | 0 (0.0%) | 0 (0.0%) | 0 (0.0%) | 0 (0.0%) | 0 (0.0%) |
| Respiratory distress, n (%) | 6 (6.7%) | 1 (1.2%) | 0 (0.0%) | 1 (0.9%) | 0 (0.0%) | 1 (1.0%) |
| Grunting, n (%) | 0 (0.0%) | 0 (0.0%) | 0 (0.0%) | 0 (0.0%) | 0 (0.0%) | 0 (0.0%) |
| Nasal flaring, n (%) | 1 (1.1%) | 0 (0.0%) | 0 (0.0%) | 0 (0.0%) | 0 (0.0%) | 0 (0.0%) |
| Head nodding, n (%) | 0 (0.0%) | 1 (1.2%) | 0 (0.0%) | 0 (0.0%) | 0 (0.0%) | 0 (0.0%) |
| Severe chest indrawing, n (%) | 3 (3.3%) | 0 (0.0%) | 0 (0.0%) | 1 (0.9%) | 0 (0.0%) | 1 (1.0%) |
| Other, n (%)^1^ | 3 (3.3%) | 0 (0.0%) | 0 (0.0%) | 0 (0.0%) | 0 (0.0%) | 0 (0.0%) |
| Very fast breathing, n (%) | 6 (6.7%) | 0 (0.0%) | 1 (1.2%) | 0 (0.0%) | 0 (0.0%) | 1 (1.0%) |
| Hypoxia (SpO_2_ < 90%), n (%) | 1 (1.1%) | 0 (0.0%) | 0 (0.0%) | 6 (5.5%) | 1 (1.0%) | 0 (0.0%) |
| Fever (≥38°C), n (%) | 3 (3.3%) | 0 (0.0%) | 1 (1.2%) | 6 (5.5%) | 1 (1.0%) | 0 (0.0%) |
| Clinically cured, n (%) | 28 (31.1%) | 58 (69.0%) | 72 (87.8%) | 7 (6.4%) | 62 (62.0%) | 86 (83.5%) |
| Clinically cured at previous visit, n (%)^2^ | -- | 23 (27.7%) | 46 (58.2%) | -- | 5 (5.1%) | 53 (54.1%) |
| Not clinically cured at previous visit, n (%)^2^ | -- | 34 (41.0%) | 23 (29.1%) | -- | 56 (56.6%) | 28 (28.6%) |
| Not clinically cured, n (%) | 62 (68.9%) | 26 (31.0%) | 10 (12.2%) | 103 (93.6%) | 38 (38.0%) | 17 (16.5%) |
| Clinically cured at previous visit, n (%)^2^ | -- | 4 (4.8%) | 6 (7.3%) | -- | 1 (1.0%) | 5 (5.0%) |
| Not clinically cured at previous visit, n (%)^2^ | -- | 22 (26.2%) | 4 (4.9%) | -- | 37 (37.0%) | 10 (9.9%) |
| ^1^ Specified as suspected pertussis syndrome, laryngeal cough, and lower limb boils | | | | | | |
| ^2^ Computed only among children with a clinically cured status available from a previous non-enrollment visit | | | | | | |
